# Cardiac magnetic resonance imaging parameters predict new-onset symptoms of heart failure in hypertrophic cardiomyopathy

**DOI:** 10.1093/eschf/xvag160

**Published:** 2026-06-09

**Authors:** Danuzia S da Silva, Vanessa Karlinski Vizentin, Iuri Ferreira Felix, Maren Maanja, Simmy A Lahori, Kalyan S Pasupathy, Steve R Ommen, Jeffrey B Geske, John R Giudicessi, Philip A Araoz, Michael J Ackerman, Adelaide M Arruda-Olson, J Martijn Bos

**Affiliations:** Department of Medicine, Mayo Clinic, Rochester, MN, USA; Department of Cardiovascular Medicine, Mayo Clinic, Rochester, MN, USA; Department of Medicine, Mayo Clinic, Rochester, MN, USA; Department of Clinical Physiology, Karolinska University Hospital, Karolinska Institutet, Stockholm, Sweden; Department of Medicine, Mayo Clinic, Rochester, MN, USA; Department of Biomedical and Health Information Sciences, University of Illinois-Chicago, Chicago, IL, USA; Department of Cardiovascular Medicine, Mayo Clinic, Rochester, MN, USA; Department of Cardiovascular Medicine, Mayo Clinic, Rochester, MN, USA; Department of Cardiovascular Medicine, Mayo Clinic, Rochester, MN, USA; Department of Radiology, Mayo Clinic, Rochester, MN, USA; Department of Cardiovascular Medicine, Mayo Clinic, Rochester, MN, USA; Department of Radiology, Mayo Clinic, Rochester, MN, USA; Department of Pediatric and Adolescent Medicine, Mayo Clinic, 200 First Street SW, Rochester, MN, USA; Department of Cardiovascular Medicine, Mayo Clinic, Rochester, MN, USA; Department of Pediatric and Adolescent Medicine, Mayo Clinic, 200 First Street SW, Rochester, MN, USA; Department of Molecular Pharmacology and Experimental Therapeutics, Windland Smith Rice Sudden Death Genomics Laboratory, Mayo Clinic, 200 First Street SW, Rochester, MN, USA

**Keywords:** Cardiac magnetic resonance imaging, Heart failure, Hypertrophic cardiomyopathy

## Abstract

**Background:**

Hypertrophic cardiomyopathy (HCM) is a heritable cardiac disorder characterized by increased left ventricular (LV) wall thickness, often leading to heart failure (HF). The role of cardiovascular magnetic resonance (CMR) imaging in predicting new onset of HF symptoms in patients with HCM remains unknown. This study aimed to identify CMR predictors of new-onset HF symptoms in individuals with HCM.

**Methods:**

This study was a single-centre retrospective cohort study of HCM patients treated at a tertiary referral centre in the USA who underwent CMR examination between 1998 and 2018, had no HF symptoms at baseline CMR, and at least 1 year of follow-up. Clinical data were collected by review of electronic medical records, and CMR images were analyzed by a blinded expert cardiac radiologis. The primary outcome was new onset of HF symptoms, defined as New York Heart Association (NYHA) class ≥ II at follow-up. Kaplan–Meier analyses and Cox proportional hazard analyses were performed.

**Results:**

Of 1462 patients diagnosed with HCM who had at least 1 CMR, 276 HCM patients without HF symptoms (average age 52.7 years, 33.3% female),median maximum left ventricular (LV) wall thickness was 19 mm ([IQR] 17–22) with a and median LV ejection fraction of 71% (IQR 66–77). Late gadolinium enhancement was detected in 56.2%) patients (60.7% had mild; 30.7% moderate; 8.6% severe). During a median follow-up period of 6.3 years, 93 patients developed HF symptoms (NYHA class II in 56 (60.2%); class III in 31 (33.3%); and class IV in 6 (6.5%). Multivariable analysis adjusted for age showed that LA enlargement (HR 1.626; 95% CI 1.01–2.62; *P* = .045) and LV mass index (HR 1.014; 95% CI 1.007–1.022; *P*≤ .001) and sex (HR 1.7; 95% CI 1.074–2.691; *P* = .023) were independent predictors of new onset of HF symptoms in patients with HCM.

**Conclusions:**

Nearly half of the patients with HCM developed HF symptoms within 6.3 years. Left atrial enlargement, LV mass index, and sex were independent predictors of new onset of HF symptoms in HCM patients. These findings emphasize the value of CMR in HF risk stratification.

## Graphical Abstract

**Graphical Abstract:**
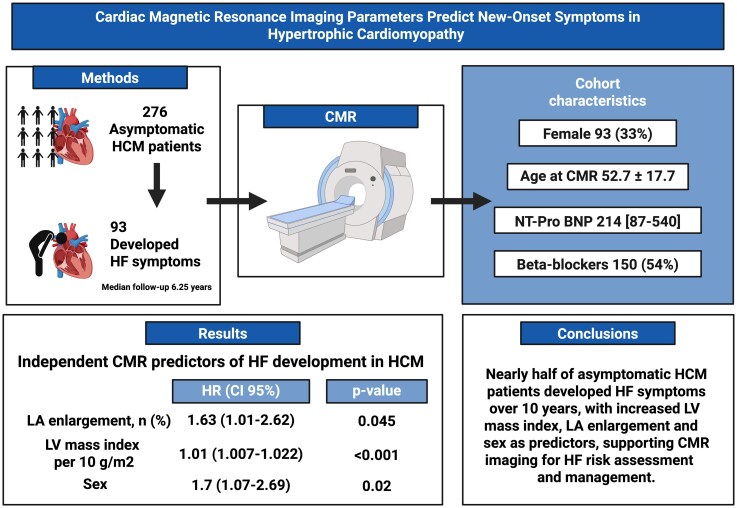
Role of CMR as a Risk Predictor for Symptom Development in HCM. Evaluation of 276 asymptomatic patients with HCM demonstrated that CMR predictors of future symptom development included severe LA enlargement, LV mass index, and sex. These findings support the utility of CMR in risk stratification and management planning for asymptomatic patients with HCM. Data are presented as mean ± SD, median (IQR), or %. HCM, hypertrophic cardiomyopathy; HF, heart failure; LA, left atrium; LV, left ventricle; CMR, cardiac magnetic resonance imaging; HR, hazard ratio; CI, confidence interval.

## Introduction

Hypertrophic cardiomyopathy (HCM) is a genetic heart condition characterized by myocardial hypertrophy without a secondary aetiology. This condition often leads to various complications such as arrhythmias, heart failure (HF), and sudden cardiac death (SCD), necessitating close monitoring and management strategies to prevent such outcomes.^1,2^

HF represents a significant burden in individuals with HCM. Progression of HF in HCM can be insidious, with symptoms ranging from dyspnoea and fatigue to overt clinical decompensation. HF in HCM is challenging to diagnose because it typically involves diastolic dysfunction without systemic congestion, making it difficult to distinguish from non-cardiac causes of dyspnoea. Furthermore, HF in HCM usually presents with preserved left ventricular (LV) ejection fraction, requiring specific approaches to risk assessment and management, as strategies for HF with reduced LV ejection fraction may not fully apply. As such, identifying the determinants and predictors of HF symptomatic development in HCM is important for patient management, timely intervention, and improvement of clinical outcomes.^3,4^

Cardiac magnetic resonance (CMR) imaging plays a pivotal role in assessing HCM, providing detailed anatomical and functional insights incremental to the echocardiography for risk assessment and therapeutic decisions.^5,6^ Previous studies have highlighted CMR's ability to detect myocardial fibrosis, correlating these findings with adverse cardiovascular outcomes and risk predictors for SCD in HCM.^7,8^ However, these studies have been limited by follow-up duration and a primary focus on end-stage outcomes including HF-related mortality, cardiac transplantation, and advanced New York Heart Association (NYHA) functional class (III or IV).

Herein, we aimed to identify CMR predictors associated with new onset of HF symptoms patients with HCM during follow-up. Elucidating the determinants of HF in individuals with HCM may allow early identification of those at higher risk for adverse outcomes, guiding personalized management strategies and shared decision-making.^9,10^

## Methods

### Study design and overview

This study was a single-centre analysis of data from an electronic health record (EHR)-based observational cohort of patients with HCM at a tertiary referral centre. Clinical and CMR data from 1998 to 2018 were collected by review of the EHR and analysis of CMR images. The study conforms to the principles of the Helsinki Declaration and was approved by the Institutional Review Board. All patients agreed to have their medical records used for research, and the Institutional Review Board waived the need for informed consent.

### Study population

For this study, patients were included if they (i) had an established, clinical diagnosis of HCM, (ii) had undergone at least one CMR at our institution, (iii) had no symptoms at baseline CMR (NYHA class I), and (iv) had at least 1 year of follow-up data available. HCM was defined as a maximum LV wall thickness ≥15 mm (or ≥ 13 mm in patients with a family history of HCM)^9,10^ Patients with known metabolic diseases (e.g. Anderson–Fabry disease), syndromic causes of HCM (e.g. Noonan syndrome), genotype positive phenotype negative for HCM, and with suboptimal CMR imaging were excluded from the study. Patients with a prior history of HF symptoms or those with prior surgical myectomy or alcohol septal ablation preceding the first CMR available were also excluded. The follow-up via EHR data was completed in December of 2023.

### Data collection

Clinical characteristics, including but not limited to history of unexplained syncope, non-sustained ventricular tachycardia, and family history of SCD were determined from EHR review. N-terminal pro b-type natriuretic peptide (NT-pro BNP) levels were collected from available records, and the institutional reference range for NT-proBNP was used, with values below 190 pg/mL considered within the normal range and values of 900–1200 pg/mL considered potentially diagnostic depending on age and kidney function. CMR data were collected for all patients from analysis of CMR data by a blinded experienced cardiac radiologist. Medications in use were collected from EHR within 1 month before or after the CMR imaging was performed, ensuring relevance to the patient's clinical status at the time of imaging. Presence apical pouch or aneurysm, dynamic LV obstruction, systolic anterior motion of the mitral valve, notable mitral regurgitation, notable left and right atrial chambers dilation, and pericardial effusion were used as categorical variables. Late gadolinium enhancement (LGE) was quantified and collected as a continuous variable. For LGE quantification, we manually segmented the myocardium excluding papillary muscles and blood pool, and subsequently selected a region of interest to represent normal myocardium. Delayed enhancement was defined using six standard deviations greater than the mean intensity within the normal myocardium.^11^ Classification of CMR findings as mild, moderate, and severe were collected based on the original classification documented in CMR reports and verified by an experienced blinded cardiac radiologist. The first CMR closest to the HCM diagnosis date was considered for analysis and designated at baseline CMR.

### Statistical methods

The primary outcome of this study was new-onset symptoms defined as NYHA class ≥II at follow-up. All statistical analyses were performed using SAS version 9.2. Variables were expressed as mean ± standard deviation, median and interquartile range (IQR), or counts and percentages as appropriate. The follow-up time for each patient was calculated from the date of their first CMR to the date of reaching the study outcome or to the date of their most recent evaluation documented in the EHR. The relationships between comparable CMR parameters were assessed by correlation analysis and employed statistical tests based on the distribution of the data. For normally distributed data, we used the Pearson correlation test and for data that did not follow a normal distribution, we performed the Kendall tau correlation test. Cox proportional-hazards regression analyses were used to identify univariable associates for new-onset HF. Effect sizes were expressed as hazard ratios (HRs) with a 95% confidence interval (CI). All CMR parameters with a two-sided *P*-value < .05 at univariable level were entered in the multivariable analysis. The cumulative probability for the occurrence of new HF symptoms was estimated using the Kaplan–Meier method.

## Results

### Study cohort

**Figure 1:**
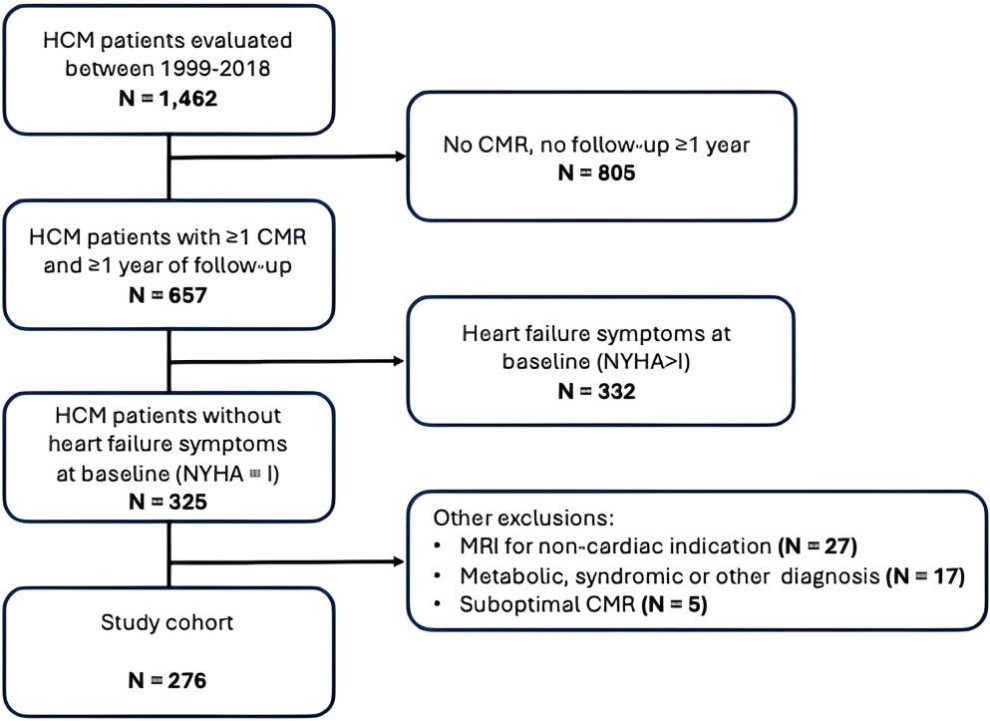
Study cohort flow chart. This flow chart depicts the selection process of the study cohort from 1462 hypertrophic cardiomyopathy patients who underwent cardiac magnetic resonance between 1998 and 2018. The final analysis focused on 276 hypertrophic cardiomyopathy patients without heart failure symptoms at baseline

A cohort of 1462 patients diagnosed with HCM with at least 1 CMR imaging study at our institution between 1998 and 2018 was reviewed. Among these, 805 patients did not have 1 year of follow-up post-CMR, reflecting the referral nature of our practice; 332 were excluded due to pre-existing symptoms at baseline (NYHA>1), 27 had undergone cardiac procedures including alcohol septal ablation or myectomy prior to CMR, 16 were excluded for concomitant metabolic or syndromic diseases, 2 were excluded because the CMR indication was analysis of the aorta or pulmonary vein study, 1 patient did not meet inclusion criteria, and for 3 patients, the CMR images were not available to review in the EHR system. After these exclusions, the study cohort consisted of 276 HCM patients without symptoms at study entry (*Figure 1*).

### Patient characteristics

Baseline characteristics of the study subjects are detailed in *Table 1*. Among the 276 eligible HCM patients, the median follow-up period was 6.3 (IQR 4.5–8.2) years. The average age of patients when CMR was performed was 52.7 ± 17.7 years with 93 (33.3%) patients female. All patients had a median N-terminal pro B-type natriuretic peptide (NT-Pro BNP) pg/mL of 213.5 [IQR 87–540]. At baseline, albeit asymptomatic, 20/190 (10.5%) of patients had a NT Pro-BNP >900 pg/mL. Chronic atrial fibrillation was present in 28 patients (10.29%), while 77 patients (28.1%) had a family history of SCD, 29 patients (10.62%) reported previous syncope, and 41 patients (15.02%) had documented non-sustained ventricular tachycardia on Holter monitoring.

**Table 1:** Hypertrophic cardiomyopathy cohort demographic characteristics

Baseline characteristics	Total cohort (*n* = 276)	Hazard ratio (CI 95%)	*P*-value
Age at cardiac magnetic resonance, mean ± standard deviation	52.7 ± 17.7	1.013 (1.000–1.025)	.050
Sex (female), *n* (%)	93 (33.3)	1.373 (.899–2.096)	.142
Atrial fibrillation, *n* (%) (*n* = 272)	28 (10.29)	1.389 (.785–2.460)	.259
Family history of sudden cardiac death, *n* (%) (*n* = 274)	77 (28.10)	.968 (.614–1.526)	.888
History of syncope, *n* (%) (*n* = 273)	29 (10.62)	1.611 (.911–2.851)	.101
Not sustained ventricular tachycardia by Holter, *n* (%) (*n* = 273)	41 (15.02)	.889 (.484–1.632)	.703
B-type natriuretic peptide per 100 pg/mL (*n* = 190) Median [interquartile range]	213.5 (87–540)	1.001 (1.000–1.001)	.009
Medications in use:			
Beta blockers, *n* (%)	150 (54.35)	1.359 (.886–2.085)	.160
Diuretics, *n* (%)	46 (16.67)	1.197 (.697–2.057)	.514
Calcium channel blockers, *n* (%)	62 (22.46)	1.070 (.668–1.714)	.778
Angiotensin-converting enzyme inhibitors, *n* (%)	41 (14.86)	.847 (.462–1.555)	.592

### Primary outcome

**Figure 2:**
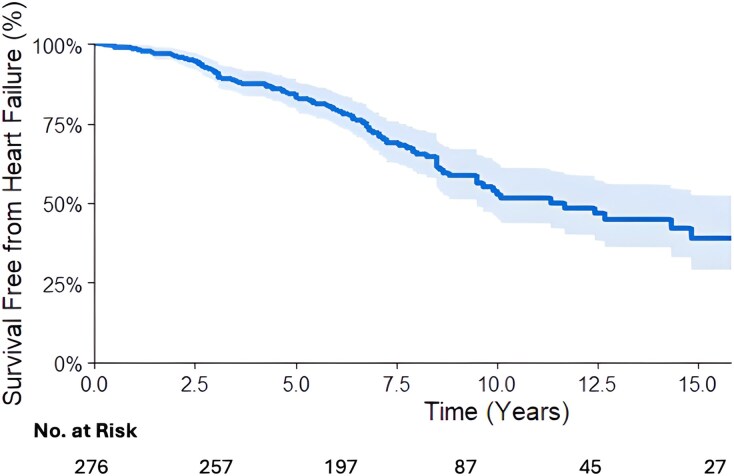
Kaplan–Meier event-free survival curve. Survival free of heart failure in the studied population. Supplemental Material: Sub-analysis of Additional Multivariate Models

During the follow-up period, 93 patients developed symptoms (NYHA class II in 56 (60.22%); NYHA class III in 31 (33.33%); and NYHA class IV in 6 (6.45%). Survival analysis estimated that 52.5% (95% CI 44.6%–61.8%) of patients remained HF symptom-free at 10 years. The Kaplan–Meier curve (*Figure 2*) shows the probability of remaining HF symptom-free over time. For treatment of obstruction, 24 patients underwent surgical myectomy during the follow-up period, and 4 patients underwent alcohol septal ablation. During the study follow-up, 21 patients died. Cause of death was available in 11, with a cardiac cause presented in three patients (one HF, one myocardial infarction, one aortic dissection).

### CMR

Baseline CMR data for the cohort are summarized in *Table 2*. Median maximum LV wall thickness was 19 mm (IQR 17–22) with LV ejection fraction of 71% (IQR 66–77). LGE was detected in 150 patients (56.2%) with quantitative assessment and described as mild LGE in 60.67%; moderate in 30.67%; and severe in 8.66%. Among the LV morphological subtypes identified in the CMR reports, the sigmoid subtype was the most prevalent, observed in 54% of patients.

**Table 2:** Hypertrophic cardiomyopathy cardiac magnetic resonance characteristics of the cohort

Cardiac magnetic resonance characteristics	Total cohort (*n* = 276)	Hazard ratio (95% confidence interval)	*P*-value
Left atrium enlargement,	171 (61.9)	1.823 (1.145–2.902)	.011
*n* (%) (*n* = 276)			
Left atrium enlargement class,			
*n* (%) (*n* = 171)			
Mild	73 (42.69)	1.034 (.657–1.628)	.884
Moderate	62 (36.25)	1.053 (.647–1.714)	.835
Severe	36 (21.05)	2.255 (1.392–3.653)	.001
Right atrium enlargement,	64 (23.18)	1.898 (1.213–2.968)	.005
*n* (%) (*n* = 276)			
Right atrium enlargement class,			
*n* (%) (*n* = 64)			
Mild	33 (51.56)	1.971 (1.129–3.441)	.016
Moderate	19 (29.69)	1.561 (.755–3.231)	.229
Severe	12 (18.75)	1.366(.554–3.367)	.498
Left ventricle end diastolic volume, per 10 ml, median, interquartile range (*n* = 275)	124.0 [99.0–154.0]	.999 (.994–1.004)	.602
Left ventricle end diastolic volume index per 10 ml/m^2^, median, interquartile range (*n* = 275)	61.58[52.0–74.0]	.995 (.983–1.007)	.439
Left ventricle end systolic volume per 10 ml, Median, interquartile range (*n* = 275)	35.0 24.0–47.0	.996 (.986–1.007)	.469
Left ventricle end systolic volume index per 10 ml/m^2^, Median, interquartile range (*n* = 275)	17.0 [13.0–23.0]	.991 (.966–1.018)	.517
Left ventricle stroke volume per 10 ml, Median, interquartile range (*n* = 275)	89.0 [71.0–108.0]	.998 (.991–1.006)	.678
Left ventricle stroke volume index per 10 ml/m^2^, Median, interquartile range (*n* = 275)	44.0 [36.0–53.0]	.997 (.980–1.014)	.718
Left ventricle mass per 10 g, Median, interquartile range (*n* = 275)	168 [132–213]	1.004 (1.001–1.006)	.017
Left ventricle mass index per 10 g/m^2^, Median, interquartile range (*n* = 275)	83.0 [69.0–100.0]	1.010 (1.003–1.017)	.004
Right ventricle end diastolic volume per 10 ml, Median, interquartile range (*n* = 275)	128.0 [102.0–157.0]	.996 (.991–1.001)	.147
Right ventricle end diastolic volume index per 10 ml/m^2^, Median, interquartile range (*n* = 275)	63.0 52.0–75.0	.987 (.974–1.000)	.054
Right ventricle end systolic volume per 10 ml, Median, interquartile range (*n* = 275)	51.0 [39.0–63.0]	.993 (.982–1.004)	.234
Right ventricle end systolic volume Index per 10 ml/m^2^, Median, interquartile range (*n* = 275)	25.0 [19.0–31.0]	.984 (.958–1.009)	.212
Right ventricle stroke volume per 10 ml, median ± interquartile range (*n* = 275)	76.0 60.0–97.0	.995 (.987–1.003)	.225
Right ventricle stroke volume Index per 10 ml/m^2^, median ± interquartile range (*n* = 275)	39.0 [31.0–47.0]	.986 (.967–1.004)	.133
Wall thickness per 5 mm, median ± interquartile range (*n* = 276)	19.00 [17.00–22.00]	1.036 (.991–1.084)	.120
Hypertrophic cardiomyopathy type, *n* (%) (*n* = 276)			
Apical	66 (23.91)	.925 (.557–1.538)	.764
Neutral	13 (4.71)	.965 (.354–2.630)	.944
Reverse curve	48 (17.39)	.726 (.416–1.269)	.261
Sigmoid	149 (53.99)	1.283 (.848–1.941)	.237
Left ventricle ejection fraction per 5%, median ± interquartile range (*n* = 275)	71.0 [66.0–77.0]	1.013 (.986–1.040)	.349
Right ventricle ejection fraction per 5%, median ± interquartile range (*n* = 275)	61.0 [55.0–66.0]	.996 (.972–1.020)	.727
Late gadolinium enhancement Percentage median ± interquartile range (*n* = 267)	.6 [.0–4.6]	1.002 (.963–1.042)	.922
Late gadolinium enhancement, *n* (%) (*n* = 267)	150 (56.18) ()	.891 (.589–1.349)	.585
Late gadolinium enhancement classification *n* (%) (*n* = 150)			
Mild	91 (60.67)	.923 (.573–1.487)	.742
Moderate	46 (30.67)	.755 (.413–1.380)	.360
Severe	13 (8.66)	1.309 (.517–3.314)	.570
Mitral regurgitation, *n* (%) (*n* = 276)	152 (55.07)	1.289 (.848–1.957)	.234
Left ventricle obstruction, *n* (%) (*n* = 276)	173 (62.68)	1.244 (.804–1.925)	.327
Pericardium effusion, *n* (%) (*n* = 276)	45 (16.30)	1.676 (.968–2.900)	.065
Mitral valve systolic anterior motion, *n* (%) (*n* = 276)	139 (50.36)	1.179 (.783–1.776)	.431
Apical pouch or aneurysm, *n* (%) (*n* = 276)	19 (6.88)	1.674 (.805–3.477)	.167

In univariate analysis, the patients who ultimately developed HF symptoms were more likely to have LA enlargement (HR 1.823 (1.145–2.902); *P*-value .011), increased LV mass (HR 1.004 (1.001–1.006); *P*-value .017) and mass index (HR 1.010 (1.003–1.017); *P*-value .004), RA enlargement (HR 1.898 (1.213–2.968); *P*-value .005) and elevated NT-pro-BNP (HR: 1.001 (1.000–1.001); *P*-value .009) at baseline CMR. Given the collinearity between LV mass and LV mass index, only the LV mass index was used in the multivariable model. Multivariable analysis adjusting for age and sex demonstrated that LV mass index (HR 1.014; 1.007–1.022, *P*-value <.001) and LA enlargement (HR 1.626; 1.010–2.620, *P*-value .045) remained independently associated with the primary outcome of new-onset HF symptoms in patients with HCM (*Table 3*).

**Table 3:** Multivariable analysis cardiac magnetic resonance adjusted by age and sex

Model variables	Hazard ratio (95% confidence interval, *P*-value)
Age	1.011 (.999–1.024, *P*-value .084)
Sex (female)	1.700 (1.074–2.691, *P*-value .023)
Left atrium enlargement *n* (%)	1.626 (1.010–2.620, *P*-value .045)
Left ventricle mass index per 10 g/m^2^	1.014 (1.007–1.022, *P*-value <.001)

## Discussion

During a median follow-up of 6.3 years, nearly half of initially asymptomatic HCM patients developed HF symptoms. These findings underscore the chronic and progressive nature of HCM and highlight the need for improved risk stratification strategies, which remain undefined in current guidelines.^10^ By incorporating data from CMR imaging, we identified that LV mass index, LA enlargement and sex were independently associated with new-onset HF symptoms at follow-up. These variables indicate that the presence of increased LV wall thickness and elevated LV filling pressures, promoting left atrial (LA) enlargement, are associated with HF in HCM patients.^12,13^ These findings emphasize the importance of earlier identification of high-risk patients, vigilant proactive monitoring and early intervention strategies to modify the risk for HF development and improve clinical outcomes in HCM patients, underscoring the growing role of CMR in the management of HCM.

While CMR was the focus of this analysis, it was worth noting that baseline levels of NT-Pro BNP were significantly higher in patients who clinically progressed, highlighting its role as an important biomarker in HCM. NT-Pro BNP is released in response to myocardial wall stress and increased ventricular pressure, conditions prevalent in HCM due to diastolic dysfunction and myocardial fibrosis. Consequently, NT-Pro BNP may serve as an early indicator of disease progression risk in HCM, offering a valuable tool for identifying patients who require closer monitoring and more proactive management to prevent the onset of symptomatic HF.^14,15^

In managing these patients, there were no differences in pharmacologic treatment strategies, such as beta-blockers, non-dihydropyridine calcium channel blockers, and diuretics, between patients who ultimately progressed to clinically evident HF symptoms and those who did not. Although these treatments are commonly used in HCM to provide symptom relief, their efficacy in altering disease progression remains inconclusive. This study did not evaluate medication doses, combinations, or duration of treatment, which could influence outcomes. The lack of significant differences in medication use underscores the need for randomized controlled trials to assess the effectiveness of pharmacological therapies and to develop new treatments for managing persistent HF symptoms in HCM.^4,16^ In addition, since patients with prior septal reduction therapies were excluded, their effects were not evaluated.

Importantly, our findings are consistent with previous literature highlighting the utility of CMR-derived parameters as predictors of different outcomes in HCM patients.^17^ LGE is a valuable tool in assessing HCM via CMR imaging, allowing visualization of myocardial fibrosis, a key feature of the pathogenesis of HCM. When present, it is classified based on its extent of the LV mass. Previous studies have shown that LGE with severe extension is associated with an increased risk of HF and SCD.^7,18^ Despite these associations, this study did not find a significant correlation between quantitative extent of LGE and the development of HF symptoms. However, the cohort with severe LGE was small (*n* = 36), which may represent a sampling bias with extensive LGE patients already symptomatic at the time of initial CMR. Therefore, future studies with larger samples and more standardized reporting and quantification of LGE on CMR are necessary to better understand the relationship between LGE and the development of HF symptoms over time and if they have concomitant progression and association.

We further identified an increased LV mass index as an independent predictor of new-onset HF symptom development in patients with HCM. This finding is consistent with the notion that LV hypertrophy, a hallmark feature of HCM, contributes to adverse cardiac remodelling predisposing individuals to HF.^19^ The progressive accumulation of myocardial hypertrophy and subsequent impairment of diastolic and systolic function may exacerbate haemodynamic compromise and precipitate HF symptoms over time. Our results emphasized the importance of assessing LV mass index on CMR as a prognostic marker, providing valuable insights into disease progression and risk stratification. In contrast, LV wall thickness, a hallmark of HCM, did not show a significant difference between those who showed clinical manifestations of HF and those who did not. This finding is particularly interesting, as greater hypertrophy is typically associated with a higher risk of adverse outcomes, as it is for SCD.^10,20^ However, the development of HF progression in HCM is likely influenced by a complex interplay of factors beyond wall thickness, including the degree of microvascular dysfunction, arrhythmic burden, and epigenetic changes. The lack of significance in this study may reflect the multifactorial nature of HF in HCM, where other factors such as LA enlargement and LV mass index, which directly contribute to diastolic dysfunction and haemodynamic compromise, play more critical roles.

LA enlargement emerged as an individual predictor of signs of HF development. LA enlargement reflects chronic pressure and volume overload, indicative of advanced myocardial and diastolic dysfunction, both common features of HCM. Dilatation of the LA is associated with increased atrial fibrosis, impaired atrial contraction, and elevated filling pressures, predisposing individuals to arrhythmias, and HF decompensation.^21,22^ LV mass index by CMR has also been previously associated with HCM-related mortality.^23^ These findings reinforce the prognostic significance of LV mass index and LA size in HCM patients, highlighting their role as surrogate markers of adverse cardiovascular outcomes and HF risk.

Despite progress in SCD risk models for HCM, additional tools are needed to identify patients at high risk for HF. Incorporating genetic data, biomarkers, multimodality imaging, and clinical variables into a comprehensive risk model could improve prediction accuracy and enable personalized management. It may also contribute to the prediction of HF outcomes in HCM patients as noted in recent studies.^24^ Such integration may also inform treatment decisions, including the use of selective cardiac myosin inhibitors, which have shown benefits in improving NYHA functional class and potentially preventing HF onset and progression.^25–27^

## Limitations

While this study provides valuable insights, it is essential to acknowledge specific limitations. The retrospective nature of our analysis introduces inherent biases and constraints associated with this study design. The relatively small size of our cohort is noteworthy. This limitation is attributed to multiple factors: exclusion of patients with baseline HF symptoms, the ongoing ramp-up in the adoption of CMR imaging in clinical practice, and loss to follow-up given the referral nature of our centre. Many patients who have received an implantable cardioverter-defibrillator likely did not undergo CMR, potentially skewing the data. The generalizability outside of our referral centre is unknown; however, the exclusion of highly symptomatic patients may better approximate a community practice. Since HF and its management are dynamic processes, a careful evaluation of each patient's timeline, including changes in CMR parameters, risk factors, and treatment effects, may provide further insight into the role of CMR in predicting HF. Future prospective studies with larger, multicentre cohorts are warranted to validate our findings and explore additional predictors of HF in HCM patients.

## Conclusions

In conclusion, nearly half of asymptomatic HCM patients developed HF symptoms within 6.3 years. Left atrial enlargement, increased LV mass index, and sex were independent predictors of new-onset of HCM-associated HF symptoms, underscoring the value of CMR in HF risk assessment and generating insights for the management of patients with HCM.

## Supplementary Material

xvag160_Supplementary_Data

## Declarations

### Data Availability

No data were generated or analysed for this manuscript.

### Pre-registered Clinical Trial Number

None supplied.
