# Supplementary material for: Cardiac magnetic resonance imaging parameters predict new-onset symptoms of heart failure in hypertrophic cardiomyopathy
Source: ESC Heart Fail. 2026 Jun 9;13(3):xvag160. doi: 10.1093/eschf/xvag160 (PMC13282896; doi:10.1093/eschf/xvag160)
Supplement: xvag160_Supplementary_Data [file xvag160_supplementary_data.docx]

**Supplemental Material: Sub-analysis of Additional Multivariate Models**

| **Model 1:** Multivariable analysis CMR adjusted by age and sex | |
| --- | --- |
| **Model variables** | **HR (95% CI, p-value)** |
| Age | 1.012 (0.994 - 1.030, p-value 0.200) |
| Sex (Female) | 1.386 (0.686 - 2.802, p-value 0.363) |
| NT-Pro-BNP | 1.000 (1.000 - 1.001, p-value 0.113) |
| LV Mass | 1.005 (1.001 - 1.009, p-value 0.020) |
| LA enlargement n (%) | 1.221 (0.656 - 2.274, p-value 0.528) |
| RA enlargement n (%) | 1.387 (0.721 - 2.669, p-value 0.326) |
| CI = confidence interval; HR = hazard ratio; LA= left atrium; LV= left ventricle; RA: right atrium. | |

| **Model 2:** Multivariable analysis CMR adjusted by age and sex | |
| --- | --- |
| **Model variables** | **HR (95% CI, p-value)** |
| Age | 1.012 (0.994 – 1.030, p-value 0.189) |
| Sex (Female) | 1.183 (0.623 – 2.248, p-value 0.608) |
| NT-Pro-BNP | 1.000 (1.000 – 1.001, p-value 0.164) |
| LV Mass Index | 1.011(1.001 – 1.021, p-value 0.024) |
| LA enlargement n (%) | 1.254 (0.674 – 2.333, p-value 0.475) |
| RA enlargement n (%) | 1.427 (0.738 – 2.758, p-value 0.290) |
| CI = confidence interval; HR = hazard ratio; LA= left atrium; LV= left ventricle; RA: right atrium | |

| **Model 3:** Multivariable analysis CMR adjusted by age and sex | |
| --- | --- |
| **Model variables** | **HR (95% CI, p-value)** |
| Age | 1.010 (0.997 - 1.023, p-value 0.135) |
| Sex (Female) | 1.561 (0.977 - 2.495, p-value 0.062) |
| LV Mass Index | 1.015 (1.007 - 1.023, p-value 0.000) |
| LA enlargement n (%) | 1.418 (0.852 - 2.360, p-value 0.179) |
| RA enlargement n (%) | 1.594 (0.957 - 2.655, p-value 0.073) |
| CI = confidence interval; HR = hazard ratio; LA= left atrium; LV= left ventricle; RA: right atrium. | |
